# A Novel Method for Real-Time Quantification of Radioligand Binding to Living Tumor Cells *In Vitro*

**DOI:** 10.1089/cbr.2022.0093

**Published:** 2024-02-13

**Authors:** Tom Bäck, Per Albertsson, Emma Aneheim, Ragnar Hultborn, Lars Jacobsson, Sture Lindegren, Stig Palm

**Affiliations:** ^1^ Department of Medical Radiation Sciences, Institute of Clinical Sciences, Sahlgrenska Academy, University of Gothenburg, Gothenburg, Sweden.; ^2^ Department of Oncology, Sahlgrenska University Hospital, Gothenburg, Sweden.; ^3^ Department of Oncology, Institute of Clinical Sciences, Sahlgrenska Academy, University of Gothenburg, Gothenburg, Sweden.

**Keywords:** binding kinetics, radioligand, flow-through microcuvette

## Abstract

**Background:**

Real-time quantification of radioligand binding to cells under *in vivo*-like conditions improves evaluation of clinical potential.

**Materials and Methods:**

SKOV-3 tumor cells were grown in a monolayer on a thin glass plate placed in a sealable shallow chamber with a continuous flow of ^125^I-trastuzumab solution. The time-dependent cell binding was measured using a NaI detector, and the binding parameters were derived by computational analysis.

**Results:**

The detection efficiency of ^125^I was 65 cps/kBq for radioligand bound to the cells. Experiments were analyzed to find the values of *k*_on_ and *k*_off_. The resulting *k*_on_ was 3.2–7.9 × 10^4^ M^−1^ s^−1^ and *k*_off_ was 0.11–4.2 × 10^–5^ s^−1^.

**Conclusions:**

Radioligands can be rapidly evaluated by binding to living cells for selection and optimization of radioconjugates for diagnostic and therapeutic purposes.

## Introduction

In the search and optimization of ligands for tumor targeting, a first evaluation often involves binding to tumor-associated purified antigens using biosensor-based techniques, such as plasmon resonance.^1–3^ For radiolabeled ligands intended for imaging or therapy, evaluation of binding properties should also include binding to living cells by detection of the radionuclide to mimic the conditions *in vivo*. Conventional cell binding assays are time-consuming and involve several manual steps. To rationalize this process, techniques that record real-time binding on living cells have been developed.^3–7^ These methods have been used for dynamic sampling to determine the type of binding (monovalent, bivalent, etc.), as well as to provide estimates of the binding kinetic parameters, that is, the rate constants of association (*k*_on_) and dissociation (*k*_off_) and the average number of receptors per cell (*B*_max_).

In this study, the authors present a novel device for real-time quantification of radioligand binding to cells. The new design allows for improved detection sensitivity, avoids cell rinsing steps, and enables rapid switching between different incubation solutions.

The aim of this study was primarily to quantitate the initial cell binding on a short time frame (hours) with a method that mimics the clinical situation of a locoregional treatment. This will assist in optimizing radioligand therapy of minimal residual disease using short-lived radionuclides, such as intraperitoneal radioimmunotherapy of ovarian cancer.^8^ The current report describes the method and the detection principle, as well as the design and performance of a prototype.

## Materials and Methods

### Basic principle

To evaluate radiation absorbed dose to tumor cells exposed to a radioligand in patients, some estimate on the kinetics with which it binds to the cells must be made. The patient situation can be simulated by exposing a cell layer to a liquid containing radioligand and measuring the cell uptake over time. Using software for dynamic simulation, the measured activity over time can then be used to find the key binding parameters that describe radioligand binding to tumor cells.

### Prototype design

**FIG. 1.**
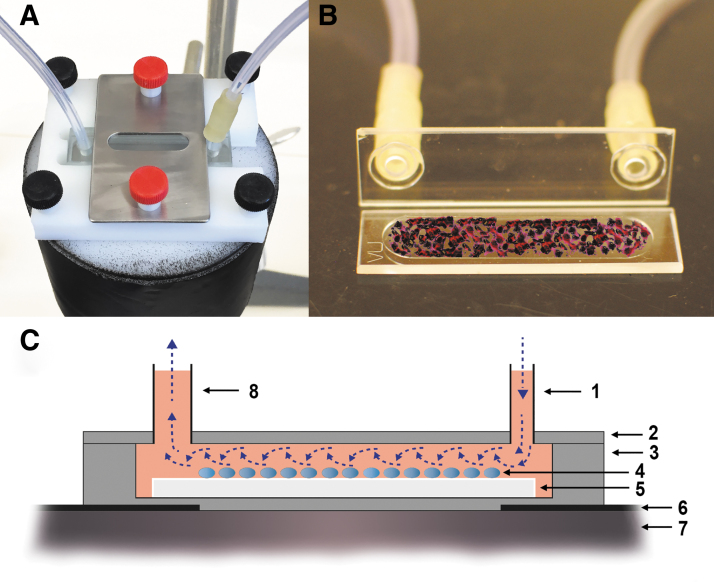
Prototype design with cuvette positioned on the detector **(A)**, glass coverslip with cells placed in cuvette chamber **(B)**, principal of the setup **(C)** with inflow (1), cuvette cover (2), cuvette (3), adhered cells (4), coverslip (5), collimator (6), radiation detector (7), and outflow (8). *Note:* Panel **(B)** and **(C)** are for illustration purposes and not scaled proportional to dimensions.

Tumor cells are grown in a monolayer on a thin glass plate, a coverslip, placed in a sealable shallow glass chamber that is exposed with a continuous flow of the radioligand solution. The chamber is mounted on top of a γ-photon detector that continuously records the radioactivity in the chamber (Fig. 1). Cell-bound radioactivity is calculated by subtracting non-bound radioactivity related to the activity concentration of the ligand solution and the chamber volume.

The radiation detector, a standard NaI(Tl) crystal (51 × 51 mm) (Metorx BV, The Netherlands), is connected to a 256-channel analyzer to generate time–activity curves for a defined energy window (Digital Ratemeter FHT 1100; Thermo Scientific, Germany). The chamber, a cuvette with internal dimensions 8 × 40 mm and 0.5 mm thickness (Type 49 Demountable Flow Through Cuvette; FireFlySci, Canada), is mounted directly on top of the detector (Fig. 1, upper left). Detector and cuvette are protected with lead to eliminate background radiation interference.

Two 20-mL syringes connected to a multisyringe pump are filled with radioligand solution or ligand-free solution and connected to the cuvette by a thin inlet tubing (Ø = 1 mm) fitted into a custom-made 6-mm thick cover cap. The glasses for cell coating (32 × 8 mm) were cut from standard 32 × 16 mm cover glasses (160-μm thick) using a glass knife and then sterilized by immersion in ethanol. Switching between the syringes was accomplished using four-port valves (Upchurch Scientific, IDEX). The outlet tubing, Ø = 2 mm, drained into a waste vial.

### Calibrations

Calibrations were performed without cells but by using similar cell medium liquid as was used for the cell uptake experiments. Detector efficiency for radiolabeled antibody in the solution was determined using a known activity concentration of ^125^I-trastuzumab. Nonspecific binding to the cuvette walls was quantitated for ^125^I-trastuzumab solutions by measuring remaining activity following a passage with nonradioactive solution. Temporary effects due to the delay when changing from nonradioactive to radioactive fluid in the cuvette were evaluated for different flows.

Cells were used to determine the detector efficiency for cell-bound activity. Efficiency was determined by collecting the coverslip with cells after an uptake experiment and measuring them in a γ-well counter (2480 Wizard^2^; PerkinElmer).

Linearity of detector response was evaluated by adding samples of ∼2 kBq ^125^I directly on the detector (covered with a thin plastic film). After each additional sample, up to a total of 10 samples, the count rate (cps) was recorded.

### Dynamic simulation

**FIG. 2.**
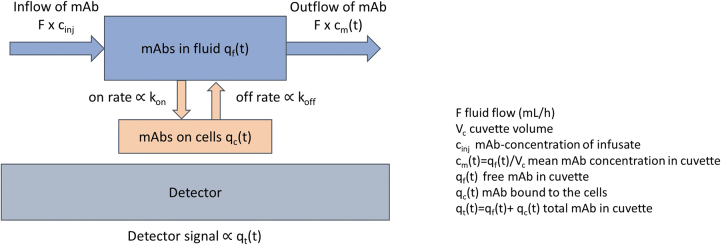
Illustration of model parameters in STELLA.

The measured kinetics was analyzed by a dynamic simulation using the software STELLA (ISEE Systems, Inc.) (Fig. 2). A program was constructed that included the following: (a) fluid flow (including change of fluids) of the apparatus; (b) antibody concentration; (c) specific activity; (d) immunoreactive fraction; (e) total amount of antigen; and (f) *k*_on_ and *k*_off_. Values for (a)–(d) were predetermined for each assay and set as fixed parameters in the program. The total amount of antigen as well as values for *k*_on_ and *k*_off_ were determined from an iterative method using least-squares fits to the net counts.

### Application for ^125^I-trastuzumab

The ovarian carcinoma cell line SKOV-3 (ATCC, Rockville, MD) was used in the experiments together with ^125^I-labeled antibody trastuzumab. The cell handling and radiolabeling of trastuzumab were performed as previously described.^9^ The immunoreactive fraction (IRF) was determined according to the study of Lindmo et al.^10^

The glass coverslips were precoated with poly-lysine (3–4 h) before one million cells were plated by pipetting four drops (4 × 25 μL) of a single-cell suspension on the centermost area of the glass. The cell concentration of the suspension was determined using an automated cell counter (NucleoCounter^®^ NC-200™; ChemoMetec A/S, Denmark) or manually by Bürker counting. The cells were plated 1–2 d before assay, and glasses were placed in petri dishes with 2 mL of cell culture medium, i.e., in RPMI 1640 medium supplemented with 10% fetal calf serum, 1% l-glutamine, and 1% penicillin–streptomycin and kept in a humidified atmosphere of 95% air/5% CO_2_ at 37°C.

Cell uptake binding kinetics was recorded for up to 3 h using 1 min per channel. The liquid flow was set at 3.2 or 6.0 mL/h. Off-rate was recorded up to 4 h during subsequent infusion of ligand-free solution.

Reproducibility was assessed by performing 17 assays (Table 1). Possible cell detachment was evaluated in separate experiments. The liquid that had passed the cell-plated cuvette with a high flow (6 mL/h) for 3 h was examined for the presence of cells.

**Table 1.** Acquisition Parameters and Output from Curve Analysis of 17 Application Runs

Assay No.	Curve analysis results		
	*k*_on_ ( × 10^3^ M^−1^∙s^−1^)	*k*_off_ ( × 10^–5^ s^−1^)	*B*_ max_*^a^* ( × 10^6^)
1	44.8	^b^	1.58
2	36.5	^b^	2.25
3	34.4	^b^	2.12
4	36.8	^b^	1.30
5	34.6	^b^	1.44
6	44.2	^b^	1.19
7	35.4	^b^	1.34
8	34.1	0.29	1.45
9	33.9	1.70	0.93
10	45.0	0.78	0.53
11	71.6	0.58	0.28
12	31.8	4.20	1.64
13	51.2	0.11	0.76
14	79.4	0.19	0.47
15	39.8	0.21	0.84
16	47.2	0.32	0.6
17	58.6	1.02	0.39
Mean	44.7	0.94	1.12
SD	13.7	1.25	0.59
CV (%)	31	132	53

^a^ Estimation of *B*_max_ was based on a cell number of 1 × 10^6^ cells, that is, the number of seeded cells.

^b^ *k*_off_ could not be estimated from analysis due to prematurely termination of acquisition time. *k*_off_ was set to 0.45, representing the median value for the derived estimations of *k*_on_.

Unspecific binding to the cells was tested by saturating the binding sites with unlabeled naked trastuzumab (20 μg/mL) for 2 h before conducting assays with ^125^I-trastuzumab.

### Comparative cell binding assay

The authors have previously published results on ^125^I-trastuzumab binding to SKOV-3 cells using a conventional method.^9^ These experiments were now repeated to provide a direct comparison with results generated by using the proposed prototype device.

### Comparative estimates of antigens per cell

The dynamic simulation provides an estimation of the total number of antigens. Since one million cells were plated for each assay, the average number of antigens per cell can be derived.

For comparison, a separate experiment was conducted. To achieve saturation, cells at low concentrations were incubated with antibodies at high concentrations. Two million SKOV-3 cells were incubated with ^125^I-trastuzumab at different antibody concentrations (2, 4, and 8 μg/mL) in a total volume of 2 mL. The incubation lasted for 4 h to allow binding equilibrium. The cells were then washed, and the cell pellets were measured for radioactivity. Results, combined with the specific activity of the radiolabeled trastuzumab, allowed the maximum number of bound antibodies per cell to be calculated.

## Results

### Calibrations

The detection efficiency of ^125^I was 65 cps/kBq for radioligand bound to the cells and 5.1 cps per kBq/mL for unbound radioligand in the solution. Unspecific binding to the cuvette walls accounted for less than 2% of the signal originating from the fluid, and less than 0.5% of that from cell-bound activity.

When changing liquids, the liquid in the cuvette will only slowly be replaced with the new liquid. Complete replacement takes ∼12 min for 3.4 mL/h and ∼6 min for a flow of 6 mL/h. This is longer than what is expected for a laminar flow, and the effect is accounted for in the dynamic simulations.

The linearity test revealed ∼2.5% dead time for the highest count rate observed in the cell uptake studies (1600 cps). When correcting for this effect on one of the uptake series, the value on *k*_on_ changed less than 1%. Therefore, dead time effects were not accounted for in the presented values for binding kinetics.

### Application for ^125^I-trastuzumab

From cell binding assays, using single-cell suspensions of SKOV-3, the IRF of ^125^I-labeled trastuzumab was found to be within an interval of 0.85–0.99 (Supplementary Fig. S1). The solution that had passed the cell-plated cuvette was analyzed for cells coming loose during the assay and the number was very low, less than 1% of the estimated number of cells plated on the cuvette. When saturating specific binding sites by first infusing cold trastuzumab (20 μg/mL) for 2 h, the subsequent cellular uptake of ^125^I-trastuzumab was reduced by 92%.

**FIG. 3.**
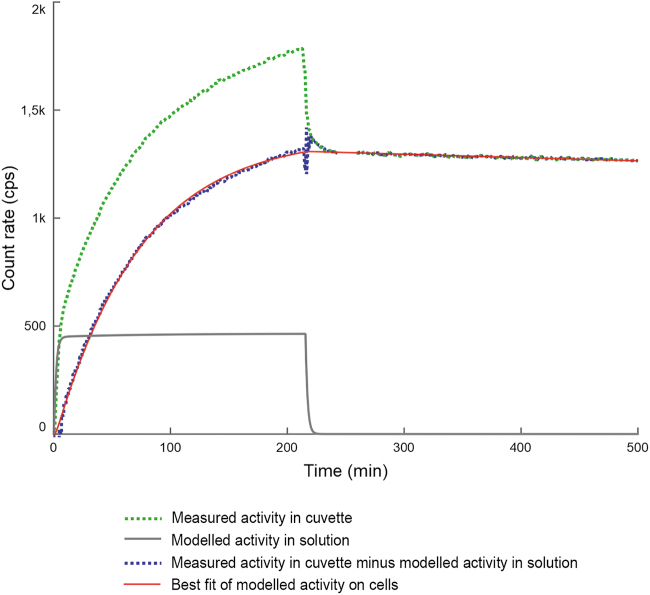
Example of the measured and corrected cell binding curves and the corresponding model fit.

The time–activity curves are for a flow of 3.2 or 6.0 mL/h and a ligand concentration of 0.5 μg/mL. The specific activity was 40–200 kBq/μg. Results from 17 different assays on cell-bound ^125^I-trastuzumab activity on OVCAR-3 cells were analyzed using the STELLA program. Figure 3 shows an example of the measured and corrected cell binding curves and the corresponding model fit.

Using an iterative comparison of the measured and modeled cell binding, the program was set to derive the values of *k*_on_, *k*_off_, and the total number of antigens. The resulting *k*_on_ was 3.2–7.2 × 10^4^ M^−1^ s^−1^ (mean 4.5; standard deviation [SD] 1.4) for an antibody concentration of 0.5 μg/mL (Table 1). The resulting *k*_off_ was 0.11–4.2 × 10^–5^ s^−1^ (mean 0.9; SD 1.2; median 0.45). By dividing the total number of antigens by the one million plated cells, the number of antigens per cell was calculated to be 0.3–2.2 × 10^6^ (mean 1.1; SD 0.6).

### Comparative cell binding assay

Using the single-cell solution assays described in the study by Palm et al.,^9^ the resulting values of the rate constants were *k*_on_ = 16.5 × 10^4^ M^−1^s^−1^ (Supplementary Fig. S2) and *k*_off_ = 2.6 × 10^–5^ s^−1^ (Supplementary Fig. S3).

### Comparative estimates of antigens per cell

Using a cell binding assay under antigen saturation conditions, the maximum number of antigens per cell was estimated to be 1.48 million per cell, that is, similar to what was observed previously (1.5 × 10^6^).^9^

## Discussion

The study group has focused on developing and optimizing α-emitters ^211^At (*t_½_* = 7.2 h) and ^213^Bi (*t_½_* = 45.6 min) targeted radionuclide therapies for minimal residual disease of disseminated cancers.^8,9,11,12^ In this effort, it is important to estimate the absorbed dose also to microtumors too small to be detected in a patient. The authors seek reliable data on the kinetics with which the ligands bind to cells. The aim of the current work was to construct a device that could generate such data in conditions that resemble those found *in vivo*. Given the short half-lives of the investigated α-emitters, the authors were particularly interested in the kinetics governing the early cellular uptakes.

In this study, the authors present a novel device where the rate with which radioligands bind (and remain) on receptors on adherent living cells can be estimated. This approach to dynamically determine the cell-bound amount has also been used by others, most notably for the LigandTracer.^13,14^ This system, combined with various assumptions on binding (monovalent, bivalent, etc.), has been used for determining *k*_on_, *k*_off_, and the number of receptors per cell. The potential advantage of the proposed prototype lies in its likely higher detector sensitivity combined with a noninterrupted measurement of the cellular uptake. This allows the use of lower activities or nuclides with shorter half-lives.

The method presented in this study generates results on the total number of antigen receptors (*N*_ar_) available for ligand binding in an assay. When *N*_ar_ is divided by the number of cells, an apparent *B*_max_ (per cell) is found. *N*_ar_ is simulated from a best fit of the pre-equilibrium data points to an expected maximum measured signal that would have resulted from following the binding reaction until the equilibrium was reached.

The accuracy of *N*_ar_ is therefore dependent on data points being collected until the curvature of the binding curve allows an estimate of its asymptotic value. In the present study, using trastuzumab on SKOV-3 cells, the authors found a minimum of 1 h to be sufficient (Supplementary Fig. S4). Since the antibody concentration is known, the rate constants *k*_on_ and *k*_off_ can also be derived from the curve fit. If *B*_max_ (per cell) and the number of cells were known for each assay, it might have improved the accuracy of the values of *k*_on_ and *k*_off_. While developing the system, the authors therefore explored different methods for estimating the number of cells, both before and after running an assay.

One method was to recover the glass after an assay, trypsinize, and count the cells in the resuspension (either manually or by cell counter). Using this method, the authors found that there was a large discrepancy between the cell number derived from cell counting and the number simulated from the model (Supplementary Fig. S5).

Another method involved retrieving the cover glass after the assay and fix and stain the cells to allow for imaging and cell counting using digital microscopy (Supplementary Fig. S6). Using this method, the difference in cell number compared with that derived from STELLA modeling corresponded to a factor of 0.5–1.7.

For both methods, it should again be noted that the actual parameter derived from the model is the total number of available antigens in the cuvette. From that number, the total number of cells was estimated using a fixed number of antigens per cell. The here assumed 1.5 × 10^6^ antigens per cell was derived from single-cell assays. Therefore, the apparent discrepancy in cell number might as well be due to differences in antigen expression for cells adhered to a surface versus that for a single-cell solution.

Analysis from phase-contrast microscopy showed that cells were growing mainly as a monolayer and that the confluency upon the experiment day was ∼75%–85% over the gross area covered by cells. For most experiments, the authors observed that the central area of the cover glass was close to 100% confluent and a small fraction of the cells were growing on top of this cell layer. Obviously, this cell density differs significantly from that in a single-cell suspension. This difference could, in turn, influence the antigen expression and how accurately it is measured. It is likely that conditions in single-cell suspensions favor antigen availability and binding. In one illustrative study, Luistro et al.^15^ measured the number of HER2 antigens on SKOV-3 cells. They used ^125^I-labeled trastuzumab on cells growing as a layer in growth plates, much like the conditions used for the real-time binding system. From Scatchard analysis, they found an average number of antigens per cell of 0.34 × 10^6^, which is a factor of 4 lower than what the authors observed using single-cell suspension. If their number of antigens per cell was used as input in the STELLA model, the estimated number of cells would decrease correspondingly, for example, from 1,000,000 to 250,000 cells. To further emphasize how these numbers vary in the literature, a markedly higher antigen expression was found by Barta et al.^16^ They reported 6 × 10^6^ antigens per cell for ^125^I-labeled trastuzumab on SKOV-3 cells in adhesion growth plates. All these observations could potentially explain the difference the authors observe in cell number between the cell counting and modeling. In addition, there is also likely a variability in the cellular antigen expression from one time to another.

For SKBR-3 cells, a cell line with a high HER2-expression level (similar to SKOV-3), a variability in the antigen expression has been observed over a 6-month period, corresponding to a threefold change in the number of antigens per cell and a coefficient of variation of up to 58%.^17^ Another mechanism that could affect the simulated number of antigens is internalization of the antibody. Internalization of trastuzumab and antigen recycling (i.e., relocation of the HER2 antigen to the cell membrane) has been observed.^18^ This phenomenon has been shown to cause changes in HER2 expression.

Overall, the attempts of the present study and those reported by others indicate that assessing the number of cells using different methods is difficult. However, since the method proposed in this study allows estimations of *k*_on_ and *k*_off_ without knowledge of the total cell/antigen number, the authors further considered that exploration in determining this number was beyond the scope of the current study.

The radiation detector used for the prototype has a high sensitivity for a wide photon energy range. This is apparent from the low noise present in the uptake curves. When comparing with preliminary results for ^125^I-mAb using the LigandTracer, the authors estimate the prototype to be ∼10 times more sensitive. Using a thicker detector would allow even higher energies to be used. This would, however, also require thicker collimators and result in lower sensitivity. On the other end, it is possible to use the device without any collimator. This increases the sensitivity but results in a lower signal-to-background ratio.

Most equipment used for the proposed prototype device can be exchanged or modified. For example, the multisyringe pump allows for syringes of different volumes. Also, cuvettes with other thicknesses are available. It is, however, recommended to keep the outlet tubing wider than the inlet to prevent pressure increase in the cuvette.

The technique mentioned in this study is based on the cuvette with cells being exposed to a flow of the radioligand solution. This flow should be sufficiently high so that the ligand concentration is not significantly reduced by ligand consumption. However, in the iterative fit of the cellular uptake to the model parameters, the effect of a reduced ligand concentration is included. Experiments gave similar results for a flow of 3 and 6 mL/h. When using a flow of 1.2 mL/h, the cell binding curve was distorted and could not be used for finding the sought parameters. A flow higher than 3 mL/h may be required for higher antigen expressions, or for higher *k*_on_, or if more cells are seeded. Higher flow may, however, result in cells being detached from the coverslips. When tested for flows up to 6 mL/h, the authors could not find any detached cells. The authors believe that the pretreatment of the coverslips with poly-lysine helps in this regard.

The authors used ^125^I-trastuzumab binding to SKOV-3 cells for a first evaluation of the prototype. The antibody concentrations were similar to that anticipated for a clinical trial on ^211^At-trastuzumab. In room temperature and using a high specific activity, the authors received values on *k*_on_ and *k*_off_ that were relatively consistent for 17 consecutive studies. The values for *k*_on_ were in line with values reported by Bondza et al. for FITC-trastuzumab on SKOV-3 cells (*k*_on_ = 3.9 × 10^4^ M^−1^ s^−1^).^14^

When using a conventional method, the authors found *k*_on_ for ^125^I-trastuzumab to be 16.5 × 10^4^ M^−1^ s^−1^, and *k*_off_ = 2.6 × 10^–5^ s^−1^. This can be compared with the previously reported^9^ *k*_on_ = 8.0 × 10^4^ M^−1^ s^−1^ and *k*_off_ = 1.2 × 10^–4^ s^−1^. All these results are in the same order of magnitude as the results achieved with the prototype.

## Conclusions

The authors present a novel device of simple construction that allow for continuous measurement of the binding of radioligands to living cells. The detector sensitivity is higher than for commercially available devices, and the ligand solution can be changed without interrupting the measurement. These advantages increase the experimental flexibility of the user.

## Supplementary Material

Supplemental data

Supplemental data

Supplemental data

Supplemental data

Supplemental data

Supplemental data
