# Supplementary material for: A Novel Method for Real-Time Quantification of Radioligand Binding to Living Tumor Cells In Vitro
Source: Cancer Biother Radiopharm. 2024 Feb 13;39(1):75–81. doi: 10.1089/cbr.2022.0093 (PMC10880261; doi:10.1089/cbr.2022.0093)
Supplement: Supplemental data [file Suppl_FigureS1.docx]

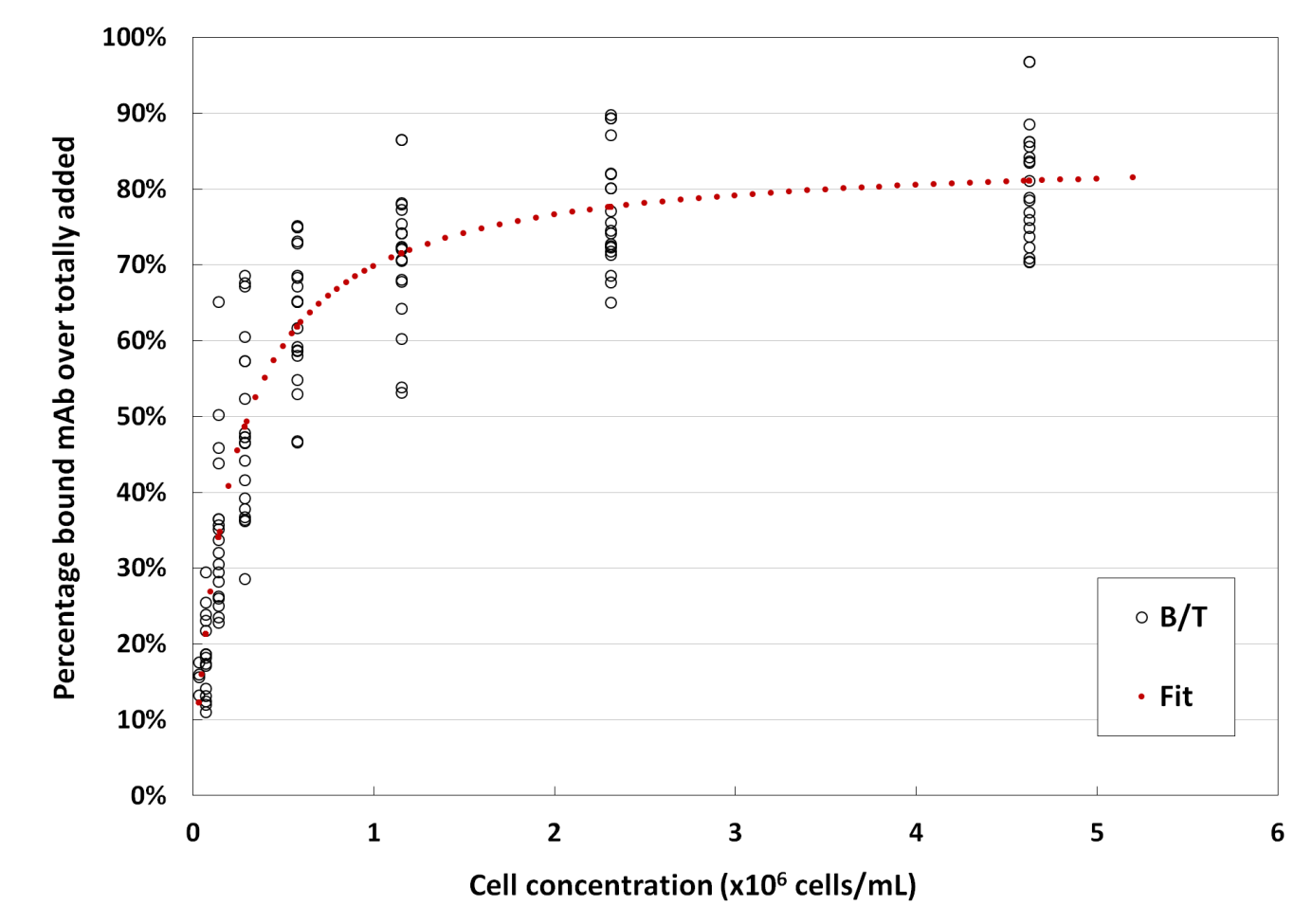


**Suppl. Fig. 1.** Compilation of all data from the conventional single-cell cell binding assays that were used for calculation of the mean IRF for all studies. The dotted curve (red) represents the curve fit from which the IRF-value was derived.
