# Supplementary material for: A Novel Method for Real-Time Quantification of Radioligand Binding to Living Tumor Cells In Vitro
Source: Cancer Biother Radiopharm. 2024 Feb 13;39(1):75–81. doi: 10.1089/cbr.2022.0093 (PMC10880261; doi:10.1089/cbr.2022.0093)
Supplement: Supplemental data [file Suppl_FigureS2.docx]

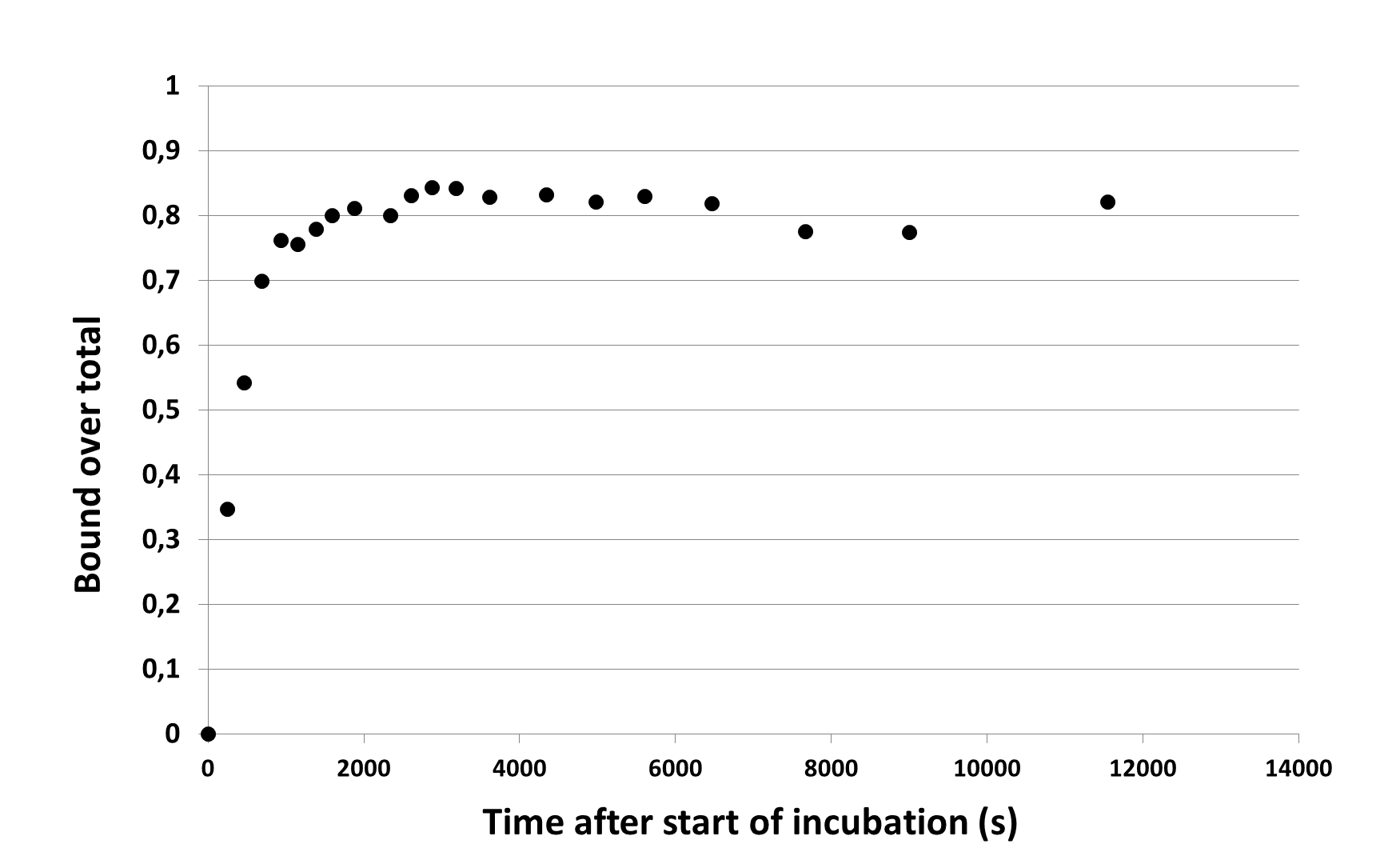


**Suppl. Fig. 2.** Binding data from the *k_on_* experiment. Fraction of cell bound antibody (bound activity over totally added) plotted as a function of incubation time.
