# Supplementary material for: A Novel Method for Real-Time Quantification of Radioligand Binding to Living Tumor Cells In Vitro
Source: Cancer Biother Radiopharm. 2024 Feb 13;39(1):75–81. doi: 10.1089/cbr.2022.0093 (PMC10880261; doi:10.1089/cbr.2022.0093)
Supplement: Supplemental data [file Suppl_FigureS3.docx]

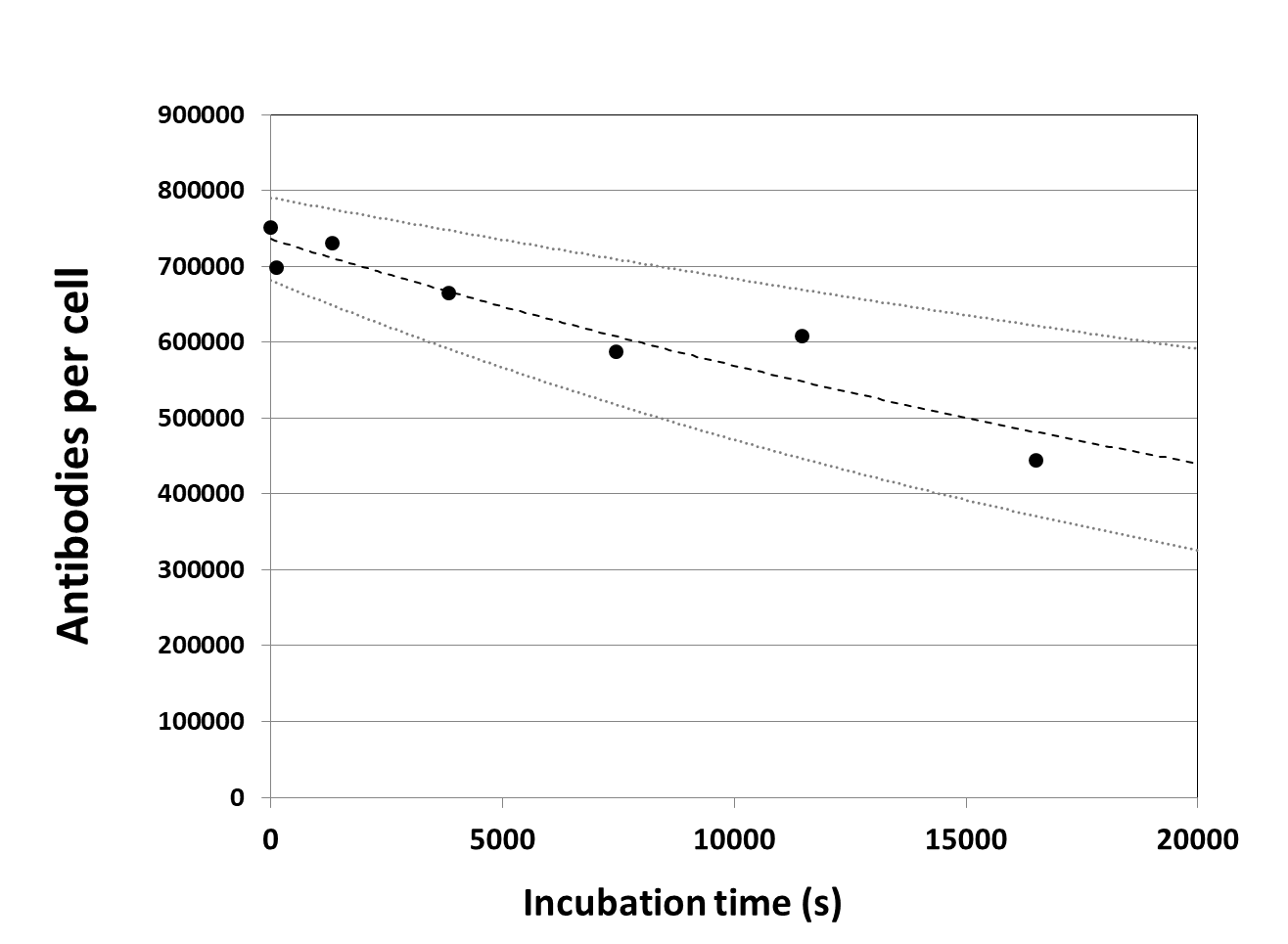


**Suppl. Fig. 3.** Binding data from the *k_off_* experiment. Number of cell bound antibodies as a function of incubation time after start of off-rate conditions. Black dashed line represents the mono-exponential fit from which the *k_off_* was derived and the grey dashed lines represent the corresponding 95% confidence interval of the curve fit.
