# Supplementary material for: A Novel Method for Real-Time Quantification of Radioligand Binding to Living Tumor Cells In Vitro
Source: Cancer Biother Radiopharm. 2024 Feb 13;39(1):75–81. doi: 10.1089/cbr.2022.0093 (PMC10880261; doi:10.1089/cbr.2022.0093)
Supplement: Supplemental data [file Suppl_FigureS4.docx]

**Suppl. Fig. 4**. Example from one assay on the time required to derive a stabilized level on k_on_ as derived from the Stella model. The one and same measured data set was used as input, with step-wise increasing number of data points.
