# Supplementary material for: A Novel Method for Real-Time Quantification of Radioligand Binding to Living Tumor Cells In Vitro
Source: Cancer Biother Radiopharm. 2024 Feb 13;39(1):75–81. doi: 10.1089/cbr.2022.0093 (PMC10880261; doi:10.1089/cbr.2022.0093)
Supplement: Supplemental data [file Suppl_FigureS5.docx]

*
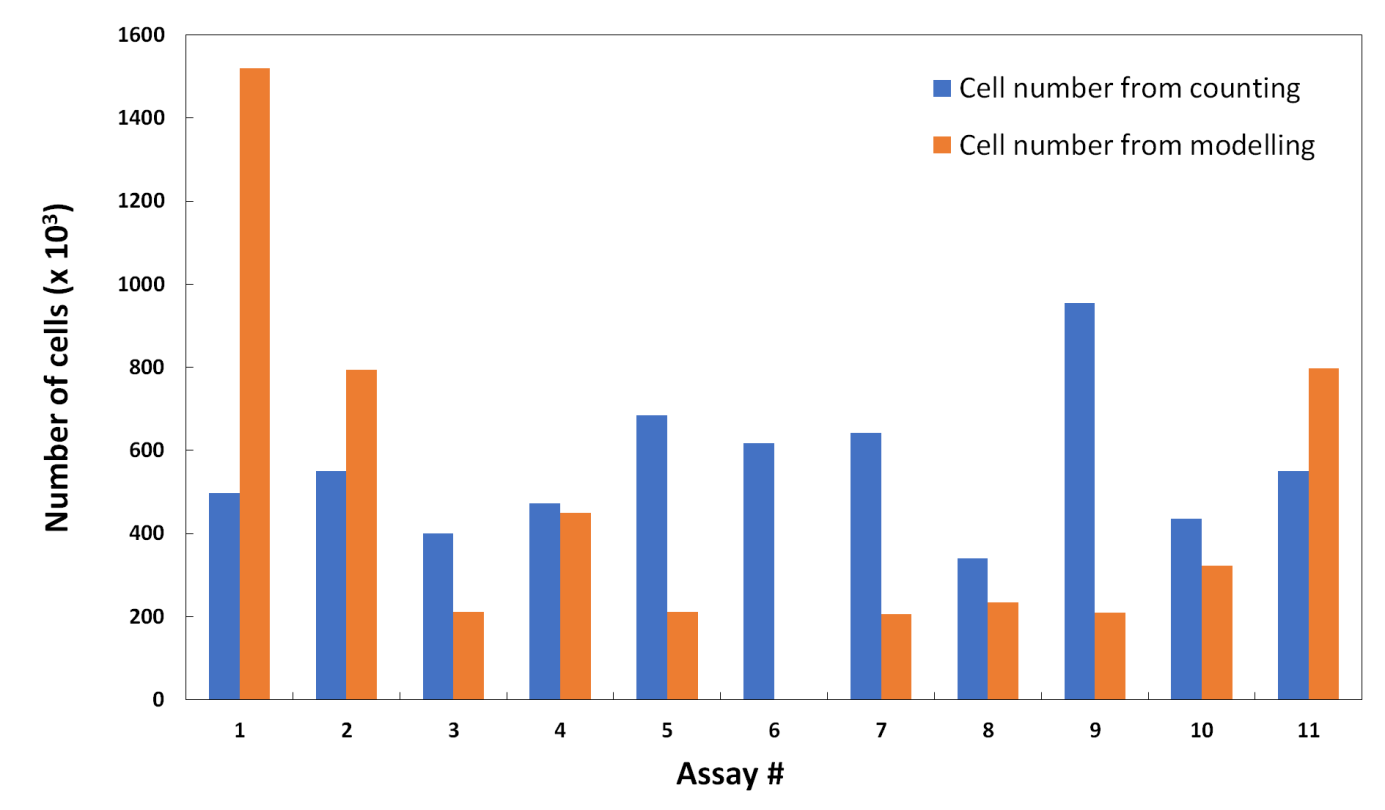
*

**Suppl. Fig. 5.** Comparison of manual cell counting (Bürker) vs cell number derived from the Stella model.
