# Supplementary material for: A Novel Method for Real-Time Quantification of Radioligand Binding to Living Tumor Cells In Vitro
Source: Cancer Biother Radiopharm. 2024 Feb 13;39(1):75–81. doi: 10.1089/cbr.2022.0093 (PMC10880261; doi:10.1089/cbr.2022.0093)
Supplement: Supplemental data [file Suppl_FigureS6.docx]

*
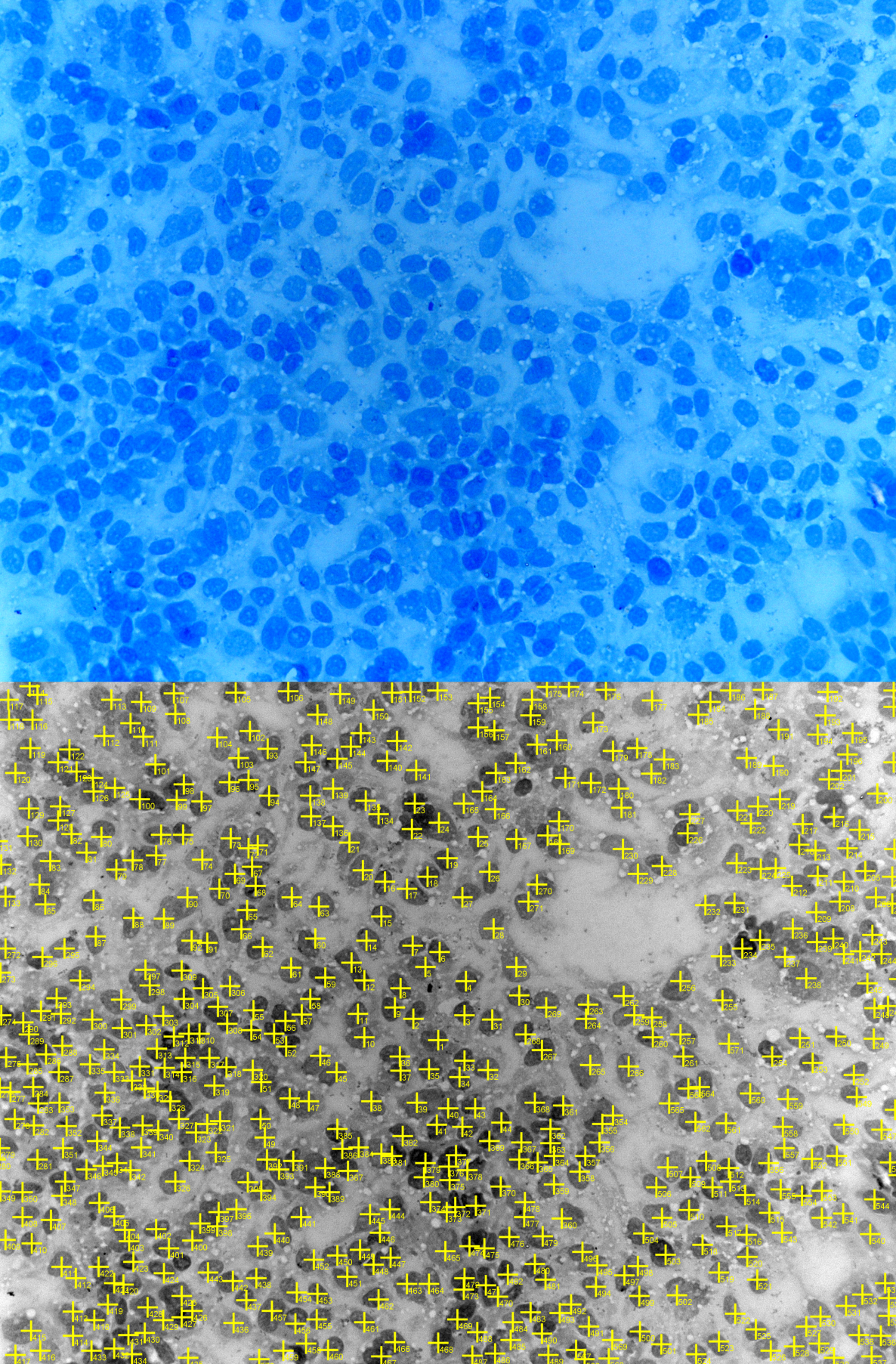
*

**Suppl. Fig. 6.** Estimation of cell number by digital microscopy. Cells were stained after an assay. The stained cover glasses were mounted on microscopy slides and imaged using a digital microscope (Nikon Coolscope). Multiple areas were selected randomly and imaged using a digital light microscope at 10x and 20x magnification. The whole cover glass was imaged using a conventional digital camera. Images from the latter allowed for quantification of the whole cell area on the cover glass from each assay. The images from microscopy allowed for counting of the total number of cells within a certain area (cells per μm^2^), or for the area void of cells (i.e. the area fraction not covered by cells), and by combining the two an estimate of the total number of cell could be made.
